# *Corynebacterium pseudotuberculosis* RNA-seq data from abiotic stresses

**DOI:** 10.1016/j.dib.2015.11.010

**Published:** 2015-11-18

**Authors:** Pablo H.C.G. de Sá, Adonney A.O. Veras, Adriana R. Carneiro, Rafael A. Barúna, Luís C. Guimarães, Kenny C. Pinheiro, Anne C. Pinto, Siomar C. Soares, Maria P.C. Schneider, Vasco Azevedo, Artur Silva, Rommel T.J. Ramos

**Affiliations:** a Institute of Biological Sciences, Federal University of Pará, Belém, Pará, Brazil; b Institute of Biological Sciences, Federal University of Minas Gerais, Belo Horizonte, Minas Gerais, Brazil; c Institute of Biological and Natural Sciences, Federal University of Triângulo Mineiro, Uberaba, Minas Gerais, Brazil

**Keywords:** Corynebaterium pseudotuberculosis, RNA-seq, SOLiD, Infection stresses

## Abstract

*Corynebacterium pseudotuberculosis* causes significant loss to goat and sheep farmers because it is the causal agent of the infectious disease caseous lymphadenitis, which may lead to outcomes ranging from skin injury to animal death (Ruiz et al., 2011) [1]. This bacterium was grown under osmotic (2 M), acid (pH) and heat (50 °C) stress and under control (Normal-BHI brain heart infusion) conditions, which simulate the conditions faced by the bacteria during the infectious process. Subsequently, cDNA of each condition was sequenced by the SOLiD3 Plus platform using the RNA-Seq technique [2], [3], [4]. The data produced was processed to evaluate the differential gene expression, which is helpful to understand the adaptation mechanisms during the infection in the host. The sequencing data of all conditions are available in the European Bioinformatics Institute (EBI) repository under accession number E-MTAB-2017.

**Specifications Table**

**Table:** 

Subject area	*Genetics*
More specific subject area	*Infectious disease*
Type of data	*Sequencing data*
How data was acquired	*SOLiD™ 3 Plus platform*
Data format	*Raw data: fastq files*
Experimental factors	*Simulation of the conditions faced by the bacteria during the infectious process (Osmotic, heat shock, acid stresses and a control condition).*
Experimental features	*We performed differentially expressed genes analysis of each stress against the control condition. After that we identified the genes that are induced and repressed among all three stresses*
Data source location	*Bahia, Brazil*
Data accessibility	*European Bioinformatics Institute. Accession number E-MTAB-2017 http://www.ebi.ac.uk/arrayexpress/experiments/E-MTAB-2017/*

## Value of the data

• This RNA-seq data allows researchers to access the transcriptomic profile of *Corynebacterium pseudotuberculosis* 1002.
• The data evaluate the use of SOLiD™ 3 Plus platform for RNA-seq experiments.
• The data helps to clarify the survival mechanisms of *Corynebacterium pseudotuberculosis* during the infectious process.

## 1 Data

The raw data files (fastq files) that were used in the analysis and interpretation in [[2], [3], [4]] are available in European Bioinformatics Institute. Accession number E-MTAB-2017. http://www.ebi.ac.uk/arrayexpress/experiments/E-MTAB-2017/.

Sample IDs are:

Transcriptoma_Coryne_1002_258_2M_1002_F3 – Osmotic stress
Transcriptoma_Coryne_1002_258_50_1002_F3 – Heat stress
Transcriptoma_Coryne_1002_258_pH_1002_F3 – Acid stress
Transcriptoma_Coryne_1002_258_N_1002_F3 – Control

## 2. Materials and methods

*Corynebacterium pseudotuberculosis* strain 1002 [1] grown in petri dishes containing BHI media (broth composed of (g/L): calf-brain infusion 200.00, beef-heart infusion 250.00, proteose peptone 10.00, dextrose 2.00, sodium chloride 5.00, di-sodium phosphate 2.50 pH 7.4±0.2 at 25 °C) at room temperature (RT). One colony was used to prepare the pre-inoculum in 20 mL of BHI media supplemented with 0.05% Tween 80. The culture was grown overnight at 37 °C in a shaker at 160 rpm. One millilitre of this pre-inoculum was used to prepare the inoculum in an Erlenmeyer flask containing 100 mL fresh BHI, and this culture was incubated at 37 °C at 160 rpm. This process was monitored until the beginning of the exponential growth phase (A600=0.2), which was reached approximately 2.5 h after the initial inoculation [3].

After the culture reached the beginning of the exponential growth phase, the inoculum was divided into 4 50-mL Falcon tubes (1 for each condition), each containing a final volume of 20 mL, and these tubes were then centrifuged for 3 min at 8000 rpm at RT. The pellet was resuspended in fresh BHI specific to each condition. For the acid stress condition, the media was supplemented with hydrochloric acid (in which the pH changed to 5). Osmotic stress was achieved with 2 M NaCl, and thermal stress was induced by resuspending the pellet in BHI medium pre-heated to 50 °C. In the control condition, bacterial pellets were resuspended in BHI medium at a physiological condition. After the addition of culture media, the tubes were kept in a shaker at 37 °C and 160 rpm for 15 min, with the exception of the thermal stress sample that was subjected to a temperature of 50 °C. An aliquot of each condition was used for decimal dilutions from 10^−1^ to 10^−6^, from which 10^−4^ to 10^−6^ bacteria were seeded in BHI agar, and petri dishes were kept at 37 °C for 48 h for viability analysis and colony counting (this step was performed in duplicate). The remaining sample was subjected to centrifugation at RT for 3 min at 8000 rpm, and the pellet was resuspended in 2 ml of RNAlater, according to the manufacturer’s instructions [3].

The bacteria suspended in RNAlater® buffer were subjected to total RNA extraction using the ChargeSwitch® total RNA cell kit (Invitrogen, USA) in accordance with the manufacturer’s recommendations, including the following adaptations: after the addition of the lysis buffer (Invitrogen), the material was transferred to 2-mL tubes partially filled with 1-mm diameter glass microbeads (Bertin Technologies). The cells were lysed mechanically using a Prescellys 24 homogeniser, set at 6500 rpm, for 2 cycles (15 s per cycle) with an interval of 30 s between the cycles. The samples were centrifuged for 1 min, and the supernatant was transferred to fresh 2-ml tubes and incubated in a dry bath at 60 °C for 15 min (represents the complete original protocol). DNase was added to eliminate the residual genomic DNA. The elution of the total RNA from the magnetic beads was performed using 100 μL of milli-Q RNase-free water. The amount of total RNA was obtained by Qubit® 2.0 fluorometer (Invitrogen) [3].

To enrich the mRNA, rRNA from each total RNA sample was removed by Ribominus™ Transcriptome Isolation kit for yeast and bacteria (Invitrogen, USA), in accordance with the manufacturer’s recommendations. The rRNA-depleted RNA was used for cDNA synthesis using the SOLiD™ Total RNA-Seq kit in accordance with the standard protocol recommended by the manufacturer, and quantification was performed by Qubit® 2.0 fluorometer (Invitrogen) [3].

The depleted RNA was fragmented using RNase III in preparation for amplification of the cDNA library, which was produced by reverse transcription from adapters attached to the ends of the RNA molecules, in accordance with the SOLiD™ Total RNA-Seq kit protocol (Life Technologies™, CA). Next, 6% denaturing polyacrylamide gel electrophoresis was performed and fragments of appropriate sizes (150 to 250 bases) were cut from the gel for cDNA amplification using PCR. Following recommended protocols, the cDNA was purified and the sizes were confirmed using 2% agarose electrophoresis. The PCR amplification in emulsion was performed using primers complementary to the adapters, in accordance with the Applied Biosystems SOLiD™ 3 Plus System Templated Bead Preparation Guide. After amplification, the microspheres were deposited onto slides for sequencing in accordance with the manufacturer’s recommendations. The SOLiD™ 3 Plus system was used to sequence the 50-nucleotide RNA reads [3].

## References

[1] Ruiz J.C., D’Afonseca V., Silva A., Ali A., Pinto A.C., Santos A.R. (2011). Evidence for reductive genome evolution and lateral acquisition of virulence functions in two *Corynebacterium pseudotuberculosis* strains. PLoS One.

[2] de Sá P.H.C.G., Veras Aa.O., Carneiro A.R., Pinheiro K.C., Pinto A.C., Soares S.C. (2015). The impact of quality filter for RNA-Seq. Gene.

[3] Pinto A.C., de Sá P.H.C.G., Ramos R.T.J., Barbosa S., Barbosa H.P.M., Ribeiro A.C. (2014). Differential transcriptional profile of *Corynebacterium pseudotuberculosis* in response to abiotic stresses. BMC Genom..

[4] Pinto A.C., Ramos R.T.J., Silva W.M., Rocha F.S., Barbosa S., Miyoshi A. (2012). The core stimulon of *Corynebacterium pseudotuberculosis* strain 1002 identified using ab initio methodologies. Integr. Biol..

